# Case series: Congenital insensitivity to pain and anhidrosis

**DOI:** 10.4103/0971-3026.40296

**Published:** 2008-05

**Authors:** Mukund D Rahalkar, Anand M Rahalkar, SK Joshi

**Affiliations:** Department of Radiology, Sahyadri Hospital, Pune, India; 1 Department of Radiology, SDMCMS and H, Sattur, Dharwad, India

Absence of pain sensation can have many etiological factors, most of which are acquired. A few congenital syndromes have been described with different clinical presentations.[1] Congenital insensitivity to pain and anhidrosis (CIPA) has a well-established genetic defect and clinical behaviour.[2] We present 2 such cases.

## Case Reports

### Case 1

**Figure 1 (A-C):**
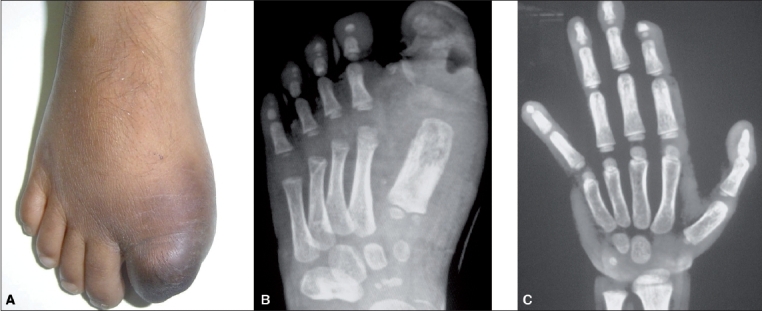
(A) Case 1: Clinical photograph of both hands showing changes of discoloration and dry gangrene of the right middle finger and the swollen finger tips. (B) Case 1: Radiograph of the left foot shows chronic osteomyelitis of the first metatarsal bone, disorganization of the metatarso-phalangeal and inter-phalangeal joints of the great toe along with irregular resorption of the phalanges, soft tissue swelling and ulceration. (C) Case 1: Radiograph of the left hand shows acrolysis of many fingers

A five-year old boy came with complaints of swelling and discharge of pus from the plantar aspect of the left great toe and the tip of the right middle finger, with progressive loss of the tips of the fingers and toes on both sides, for the last two years [Figure 1A]. There was a history of consanguineous marriage with a normal sibling.

On examination, the patient had swelling, changes of dry gangrene, discoloration and ulceration involving both great toes and the tips of multiple fingers and toes, along with cellulitis. The peripheral pulses were normal. The radiographs of the feet and hands showed changes of acrolysis, osteomyelitis and soft tissue ulcer [Figure 1B and C]. The nerve conduction velocity was reported as sensorimotor neuropathy, severe in both limbs. There was loss of hot and cold sensation in the lower halves of all limbs.

Sural nerve biopsy showed a reduced number of unmyelinated and small myelinated fibers. No granuloma was found. It was noticed that that the child did not feel any pain during injection of the anesthetic.

### Case 2

**Figure 2:**
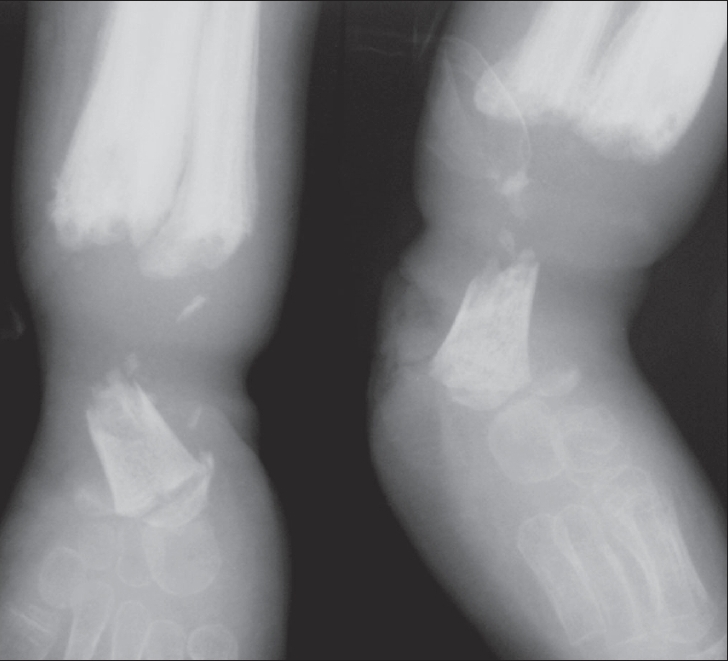
Case 2: Radiograph of the left ankle shows chronic osteomyelitis with periosteal reaction of the first metatarsal bone (arrow) and the lower ends of the tibia and fibula. The latter reveal marked destruction with fracture non-union of tiny sequestra

A five-year old boy came with complaints of eating his own lips, tongue and fingers without feeling any pain. He did not sweat since birth and had a history of high fever at the age of four months. He had a non-healing wound over the right foot since birth and on examination showed mutilation of all fingers, right great toe and the first to the fourth toes, a torn and healed right ear lobe, a wound over the right knee with maggots and fractures of the lower ends of the tibia and fibula with osteomyelitis and sequestration on the left [Figure 2]. A biopsy of the discharging sinus over the ankle showed chronic osteomyelitis.

## Discussion

Hereditary sensory and autonomic neuropathy (HSAN) is a rare syndrome characterized by congenital insensitivity to pain and temperature changes and by autonomic nerve formation disorder, as described by Dua.[1] HSAN has five types. Type IV is inherited as an autosomal recessive disorder due to lack of maturation of small myelinated and un-myelinated fibers of the peripheral nerves, which convey sensation of pain and temperature.[1] This type IV is also called CIPA.

CIPA, which was first described in 1951, results from a defect in neural crest differentiation and the system responsible for pain and temperature sensation, the first order afferent system. Ultrastructural and morphometric studies of the peripheral nerves reveal loss of unmyelinated and small myelinated fibers and no innervation to the sweat glands. These features suggest that a defect in the differentiation and migration of neuronal crest elements and possible degradation of the nerve growth factor/neurotrophic tyrosine receptor kinase 1 (NGF/NTRK 1) pathway may be responsible for CIPA. Mutations within its tyrosine kinase pathway leading to such degradation have been discussed by Greco *et al.*[2]

Though CIPA is a very rare disorder, more than 300 cases have been reported from Japan, with about 60 cases reported from the United States of America.[3]

CIPA usually manifests in childhood, with repeated, unrecognized trauma and resultant self mutation. Most of the affected children also show mental retardation.Due to painless injuries, the bones, joints and soft tissues of the extremities as well as the orbits, nasal cavities and oral cavity undergo mutilating effects, for which the parents seek medical attention and treatment.[4] Bar-On *et al.*,[4] have also described preventive measures for orthopaedic complications such as use of special shoe ware, periods of non weight-bearing, surgical wide debridement and curative osteotomy for deformity.

E Bar-On *et al.* have also added a few more investigations in the total work-up of CIPA patients, for the sake of accurate diagnosis, which include a quantitative sweat test and an intra-dermal histamine test to check for anhidrosis, along with DNA studies to look for specific mutations. Seyon *et al.*,[5] have called CIPA, the “mystery of broken bones” after the case of a 15-years-old boy who was wrongly labeled as a case of osteogenesis imperfecta due to recurring fractures since the age of 4.

As in our second patient, dental and oral cavity complications, including tooth loss (auto-extraction), bitten tongue, ulceration and lip injury are also known.[6] Use of mouth guard and early tooth extraction are described to prevent these complications. Skin complications due to anhidrosis[7] and ocular complications due to painless injuries[8,9] are not uncommon.

Both patients had loss of temperature sensation and episodes of high fever. Overheating has in fact been described in the literature to kill more than half of the number of children by CIPA before the age of 3.[3]

These two cases amply highlight many of the complications of CIPA.
